# Efficient Tomato Disease Detection Using MaxMin-Diffusion Mechanism and Lightweight Techniques

**DOI:** 10.3390/plants14030354

**Published:** 2025-01-24

**Authors:** Haoxin Guo, Jiarui Liu, Yan Li, Yifei Xu, Keyi Xu, Anzhuo Fan, Jiarui Hao, Yifei Hou, Chunli Lv

**Affiliations:** 1 China Agricultural University, Beijing 100083, China; 2 Beijing Forestry University, Beijing 100083, China

**Keywords:** tomato disease detection, smart agriculture, machine learning, time-series model, lightweight model deployment

## Abstract

This paper proposes a disease detection model based on the maxmin-diffusion mechanism, aimed at improving the accuracy and robustness of disease detection tasks in the agricultural field. With the development of smart agriculture, automated disease detection has become one of the key tasks driving agricultural modernization. Traditional disease detection models often suffer from significant accuracy loss and robustness issues when dealing with complex disease types and dynamically changing time-series data. To address these problems, this paper introduces the maxmin-diffusion mechanism, which dynamically adjusts attention weights to enhance the model’s focus on key disease regions while suppressing interference from irrelevant areas, significantly improving the segmentation accuracy of disease regions. Through a series of experiments, the proposed model demonstrates outstanding performance across various disease detection tasks. For bacterial spot disease detection, the model achieves a precision of 0.98, recall of 0.95, accuracy of 0.96, and mIoU of 0.96, indicating that it can efficiently and accurately identify disease regions even in complex backgrounds. Compared to traditional self-attention and CBAM mechanisms, the maxmin-diffusion mechanism shows significant advantages in fine-grained feature extraction and time-series data processing, particularly in the recognition of dynamically changing disease regions, where it exhibits higher detection accuracy and robustness. Furthermore, the model underwent lightweight optimization, enabling the proposed disease detection model to not only achieve high-precision detection but also run efficiently on resource-constrained mobile devices. This provides strong technical support for the application of smart agriculture.

## 1. Introduction

Agricultural production faces significant challenges due to global climate change and the increasing severity of plant diseases and pests [1,2,3,4,5]. In the northwestern region of China, Bayannur is an essential agricultural hub with extensive tomato cultivation and high economic value. However, tomatoes are highly susceptible to various diseases during their growth cycle, including fungal, bacterial, and viral infections [6]. These diseases not only reduce crop yield and quality [7] but also threaten farmers’ income and food safety [8,9,10]. The limitations of traditional manual disease detection methods, such as significant labor and time requirements and high susceptibility to human error, further exacerbate the challenges of effective disease management [11,12].

Tomato cultivation, characterized by its short growth cycle and high vulnerability to environmental factors, especially benefits from rapid and precise disease detection systems. These systems enable the real-time monitoring of farmland, timely identification and diagnosis of diseases, and provision of scientific decision support for disease management [13,14]. These advancements are instrumental in enhancing agricultural productivity, ensuring healthy crop growth, reducing environmental pollution, and improving food safety [15]. Without early detection and effective intervention, the rapid spread of diseases during tomato growth can lead to significant losses, making the development of precise and efficient detection systems essential for protecting yields and farmers’ incomes [16,17,18].

Recent advancements in deep learning have significantly expanded the application of computer vision in agricultural disease detection [19,20,21]. Several approaches have achieved promising results in tomato leaf disease detection, particularly with static images. For instance, Rahman et al. proposed a system based on support vector machines (SVMs) for the automated detection, identification, and treatment of tomato leaf diseases, achieving an accuracy exceeding 85% [22]. Zhou et al. introduced a novel deep learning model—the Reconstructed Deep Residual Dense Network—that efficiently extracts meaningful features from images, attaining a 95% accuracy in tomato leaf disease identification tasks [23]. Agarwal et al. improved convolutional neural network (CNN) models by adding convolutional and pooling layers and adopting batch normalization, achieving a 98.4% accuracy in tomato leaf disease identification [24]. Furthermore, a modified YOLOv3 algorithm incorporating multi-scale feature detection and object bounding box clustering demonstrated a detection accuracy of 92.39% with a detection time of only 20.39 ms [24]. Altuntacs et al. integrated deep features from AlexNet, GoogLeNet, and ResNet-50 models to train an SVM classifier, achieving a classification accuracy of 96.99% for tomato leaf disease images [25]. While these methods achieve high accuracy for static image analysis, they lack the capacity to capture temporal features critical for understanding disease progression in real-world agricultural scenarios.

In practical agricultural scenarios, the growth of tomatoes and the progression of diseases are dynamic processes, making static image analysis insufficient for capturing temporal disease development. To address these limitations, a novel tomato disease segmentation model is proposed in this study, incorporating the maxmin-diffusion module and time-series segment network. The main contributions of the proposed model are outlined as follows:

- Maxmin-diffusion module: The proposed module uses maximum–minimum diffusion to enhance attention on anomalous regions, suppress background noise, and improve segmentation accuracy, even in complex contexts.
- Temporal models: A time-series network integrates spatial and temporal features, enabling the model to capture disease dynamics and leverage historical data for improved prediction accuracy.
- Maxmin loss function: This loss function assigns weights to diseased and background regions, enhancing segmentation precision, reducing false positives, and focusing on critical temporal frames.
- Deployment in Bayannur: The model, trained on locally collected tomato video data, shows strong generalization and supports real-time, precise disease monitoring and control.

In summary, the proposed maxmin-diffusion and time-series model significantly improves segmentation accuracy and effectively handles dynamic disease progression. The model’s application to tomato disease detection in Bayannur provides real-time, precise support for agricultural production and contributes to the advancement of intelligent agriculture.

## 2. Related Work

### 2.1. Diffusion Models

Diffusion models, particularly Denoising Diffusion Models (DDMs) [26], have achieved notable success in generation tasks in recent years. These models simulate a stochastic process to progressively restore the original data distribution from an initial state of Gaussian noise [27]. The core concept of diffusion models involves the gradual addition and removal of noise to enable data generation or recovery. In the domain of image generation, diffusion models have demonstrated exceptional performance in terms of image quality and detail restoration. The mathematical formulation of the diffusion process can be described in two stages:

1. Forward diffusion process: Noise is incrementally added to data point $x_{0}$ until it is transformed into complete Gaussian noise. The forward process is represented as
  (1) $$q\left(x_{t}|x_{t-1}\right)=N\left(x_{t};\sqrt{1-\beta_{t}}x_{t-1},\beta_{t}I\right),$$
  where $x_{t}$ denotes the data after *t* steps of noise diffusion, $\beta_{t}$ represents the noise intensity at each stage, and N indicates a Gaussian distribution.
2. Reverse diffusion process: The original data are reconstructed step-by-step from Gaussian noise. The reverse process is expressed as
  (2) $$p_{\theta }\left(x_{t-1}|x_{t}\right)=N\left(x_{t-1};\mu_{\theta }\left(x_{t},t\right),\sigma_{\theta }\left(x_{t},t\right)\right),$$
  where $\mu_{\theta }\left(x_{t},t\right)$ and $\sigma_{\theta }\left(x_{t},t\right)$ represent the predicted mean and standard deviation, learned by network parameter θ.

The diffusion mechanism restores data to their original distribution by simulating both the forward and reverse processes. Diffusion models are particularly effective in generation tasks due to their ability to learn fine-grained data patterns and extract these patterns during the generation process [28]. For example, in image generation, diffusion models can recover details from blurry images through iterative denoising, producing natural and clear results. The advantages of diffusion mechanisms extend beyond image generation to feature extraction and fine-grained pattern representation [29]. Traditional CNNs often rely on local feature extraction, which can result in significant information loss, especially when processing images with complex details and textures [30]. By employing global noise propagation and denoising operations, diffusion models effectively capture global image features while progressively enhancing detail information, providing refined feature representations for subsequent segmentation tasks. In this study, the maxmin-diffusion module leverages the strengths of diffusion mechanisms to enhance fine-grained features in diseased tomato regions, improving the accuracy of segmentation in these areas.

### 2.2. Time-Series Models

Time-series models [31], particularly Long Short-Term Memory (LSTM) networks [32], Gated Recurrent Units (GRUs) [33], and Transformer models [34], have been widely applied in dynamic feature learning tasks. These models exhibit strong capabilities in video analysis, semantic segmentation, and time-series data prediction. Unlike static images, video data inherently include temporal characteristics, and the relationships between frames provide critical cues for content analysis. In agricultural videos, disease progression is a dynamic process, typically evolving under specific conditions. Capturing temporal variations in disease expansion is crucial for accurate detection. LSTM and GRU models, as traditional variants of Recurrent Neural Networks (RNNs), address the vanishing and exploding gradient problems encountered in standard RNNs during long-sequence training. LSTM incorporates gating mechanisms (input gate, forget gate, and output gate) to regulate information flow effectively, enabling the network to learn long-term dependencies. The mathematical formulation of LSTM is as follows:

(3) $$f_{t}=\sigma \left(W_{f}x_{t}+U_{f}h_{t-1}+b_{f}\right),$$

(4) $$i_{t}=\sigma \left(W_{i}x_{t}+U_{i}h_{t-1}+b_{i}\right),$$

(5) $$o_{t}=\sigma \left(W_{o}x_{t}+U_{o}h_{t-1}+b_{o}\right),$$

(6) $$c_{t}=f_{t}⊙c_{t-1}+i_{t}⊙\tanh\left(W_{c}x_{t}+U_{c}h_{t-1}+b_{c}\right),$$

(7) $$h_{t}=o_{t}⊙\tanh\left(c_{t}\right),$$

where $f_{t}$, $i_{t}$, and $o_{t}$ are the forget, input, and output gates, respectively; $c_{t}$ represents the cell state; $h_{t}$ is the output; σ denotes the Sigmoid activation function; and ⊙ indicates element-wise multiplication. LSTM captures the evolution of temporal data by effectively memorizing and updating sequential context relationships.

Transformer models have demonstrated superior performance in processing long-sequence data, particularly in handling large-scale datasets. Transformers leverage self-attention mechanisms to capture long-range dependencies and enable parallel computation, significantly enhancing training efficiency. The self-attention mechanism is mathematically defined as

(8) $$\mathrm{Attention}\left(Q,K,V\right)=\mathrm{softmax}\left(\frac{QK^{T}}{\sqrt{d_{k}}}\right)V,$$

where *Q*, *K*, and *V* represent query, key, and value matrices, and $d_{k}$ is the key dimension. Through the dynamic adjustment of attention weights based on the relative importance of input sequence elements, self-attention mechanisms enhance the modeling of long-range dependencies. In tomato disease detection, incorporating time-series models helps capture the temporal evolution of diseases, enhancing the ability to predict diseased regions [35]. Through the integration of a time-series network, spatial features from individual frames are combined with temporal information from preceding and succeeding frames, enabling more accurate segmentation of diseased regions. The application of time-series models significantly enhances the capability to learn dynamic features in agricultural video data, adapting more effectively to the progressive nature of tomato diseases.

### 2.3. Semantic Segmentation

Semantic segmentation, a fundamental task in computer vision, aims to classify every pixel in an image into specific categories [36]. In agricultural disease detection, semantic segmentation is utilized to classify tomato images at the pixel level, accurately identifying diseased regions. Advances in deep learning, particularly CNNs, have led to the development of several seminal semantic segmentation models, such as U-Net, DeepLab, and PSPNet (Pyramid Scene Parsing Network). The U-Net model introduced an encoder–decoder structure that combines contextual information with local details to achieve accurate segmentation [37]. Its core concept lies in using skip connections to merge low-level features from the encoder with high-level features from the decoder, improving segmentation precision. The U-Net structure is expressed as

(9) $$y=f_{\mathrm{decoder}}\left(f_{\mathrm{encoder}}\left(x\right)\right),$$

where *x* denotes the input image, *y* represents the segmentation output, $f_{\mathrm{encoder}}$ indicates the feature extraction process, and $f_{\mathrm{decoder}}$ refers to the reconstruction process. This architecture preserves high-resolution details, achieving outstanding performance in segmentation tasks. The DeepLab series (DeepLabv1-v3+) integrate dilated convolutions and Conditional Random Fields (CRFs) to optimize segmentation boundaries, significantly improving boundary precision [38]. Dilated convolutions expand the receptive field without increasing computational complexity, enabling the extraction of larger contextual information. In DeepLabv3+, dilated convolutions are combined with the Atrous Spatial Pyramid Pooling (ASPP) module, enhancing multi-scale feature extraction. The ASPP operation is represented as

(10) $$y=\mathrm{ASPP}\left(x\right)=\left[{\mathrm{Conv}}_{1\times 1}\left(x\right),{\mathrm{Conv}}_{3\times 3}\left(x,d=6\right),{\mathrm{Conv}}_{3\times 3}\left(x,d=12\right),\ldots \right],$$

where ${\mathrm{Conv}}_{k\times k}$ denotes a k×k convolution operation, and *d* represents the dilation rate. This approach effectively extracts multi-scale information, improving segmentation accuracy. In tomato disease detection, traditional semantic segmentation methods face challenges such as varying lighting conditions, occlusions, and complex backgrounds [39]. Deep learning methods overcome these challenges by efficiently extracting relevant features tailored to agricultural scenarios. This study combined diffusion models and time-series networks to provide a more precise solution for segmenting diseased tomato regions, addressing the limitations of conventional approaches.

## 3. Materials and Methods

### 3.1. Materials

#### 3.1.1. Dataset Collection

**Figure 1:**
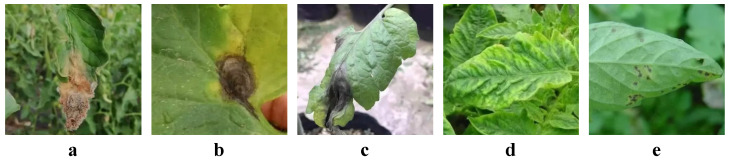
Dataset samples: (**a**) is gray mold, (**b**) is early blight, (**c**) is late blight, (**d**) is viral disease, and (**e**) is bacterial spot disease.

In this study, the collection of the dataset formed the foundation for training the tomato disease detection model. To ensure the diversity and comprehensiveness of the dataset, we collected a large number of videos from tomato cultivation areas in Linhe District, Bayannur City, Inner Mongolia, and supplemented these with videos from the internet. The dataset consisted of annotated samples for five common tomato diseases, including gray mold, late blight, early blight, viral diseases, and bacterial spot disease. The total number of collected images for each disease is presented in Table 1. As shown in Figure 1, the dataset included representative images for each disease, covering various disease stages and symptom manifestations.

The data collection primarily took place in tomato planting areas in Linhe District, Bayannur City, Inner Mongolia. This region, characterized by arid climate, large temperature fluctuations, and extensive tomato cultivation, exhibits typical agricultural disease types, making it an ideal source for tomato disease samples. During the collection process, we selected multiple cultivation bases to ensure that the disease characteristics of tomatoes under different environmental conditions were adequately represented. We particularly focused on different growth stages of the tomato plants, including the seedling stage, fruiting stage, and aging stage, as the disease symptoms presented at each stage may vary. Regarding the equipment and techniques used for image collection, we employed a high-resolution digital single-lens reflex camera with a resolution of 6000×4000 pixels, ensuring image clarity and detailed representation. To maintain consistency in shooting angles and lighting conditions, we equipped the setup with a stable tripod. Additionally, to improve the efficiency and accuracy of image collection, we used different shooting angles, including front, side, and diagonal angles, to comprehensively capture disease features on the plant’s leaves, stems, and fruits. During the collection process, we paid particular attention to diagnostic markers of the diseases. For example, we ensured that the dataset captured early, intermediate, and advanced stages of each disease to enhance the model’s ability to detect and classify various symptom levels.

The characteristic differences between the various diseases were significant and essential for training the computer vision model. For gray mold, the disease is characterized by a gray mold layer on the surface of the plant leaves, typically expanding from the leaf edges inward and forming water-soaked, damp spots. As the disease worsens, the mold spreads across the entire leaf, causing the leaf to soften and decay. Late blight manifests as dark green to black spots on the leaves, with fuzzy edges and irregular shapes, typically with a water-soaked center. As the disease progresses, the spots enlarge, the leaves yellow, and the plant begins to shrink. Early blight is characterized by small yellow-brown spots on the leaves, often with purple or red edges. As the disease intensifies, the spots expand, causing widespread wilting. Viral diseases result in deformed, yellowing leaves, with some leaves showing mottled patterns of yellow or white irregular patches. Bacterial spot disease shows small water-soaked spots on the leaves, which turn yellow at the edges as the disease progresses, ultimately causing the leaves to dry up.

#### 3.1.2. Video Enhancement

To improve video quality and enhance the accuracy of subsequent models, various enhancement techniques were applied during the preprocessing phase, including denoising, contrast enhancement, and color correction. Noise is a significant issue in agricultural videos due to environmental lighting variability and equipment limitations. Gaussian filtering was utilized to remove noise. By performing weighted averaging on each pixel, Gaussian filtering effectively reduced high-frequency noise while preserving image smoothness. The Gaussian filter is mathematically defined as

(11) $$G\left(x,y\right)=\frac{1}{2\pi \sigma^{2}}e^{-\frac{x^{2}+y^{2}}{2\sigma^{2}}},$$

where G(x,y) represents the Gaussian filter response at position (x,y), and σ is the standard deviation of the filter, controlling its smoothing degree. This filter averages pixel values based on surrounding pixels, thereby removing noise and retaining essential details.

Contrast enhancement aimed to improve the visibility of diseased regions, making them more discernible against complex backgrounds. Histogram equalization, a common contrast enhancement technique, was employed to adjust the grayscale distribution of the image, effectively enhancing its contrast. The histogram equalization process is represented as

(12) $$s=T\left(r\right)=\frac{L-1}{M\cdot N}\sum_{i=0}^{r}h\left(i\right),$$

where *s* represents the output grayscale value, *r* denotes the input grayscale value, *L* is the number of gray levels, *M* and *N* are the image width and height, respectively, and h(i) is the frequency of grayscale value *r* in the input image. By redistributing grayscale values, this technique highlights low-contrast regions, making variations in diseased areas more apparent. Color correction was applied to mitigate chromatic deviations caused by lighting conditions and equipment differences, which often obscure the visibility of diseased regions. Basic color correction methods included white balance adjustment and color temperature tuning. White balance adjustment balanced the image’s color composition, aligning it with natural light conditions. The white balance adjustment process is defined as

(13) $$I_{wb}\left(x,y\right)=\frac{I(x,y)}{W},$$

where I(x,y) represents the pixel value at position (x,y) in the input image, and *W* is the white balance coefficient. This adjustment calculated and corrected chromatic deviations in each pixel, aligning the color temperature across the image and eliminating lighting-induced bias. The process enhanced the visibility of diseased regions under varying lighting conditions.

Through the implementation of these video enhancement techniques, noise and unfavorable lighting conditions in the raw videos were effectively mitigated, rendering diseased regions in tomato plants clearer and more distinct. These enhancements provided high-quality input data for subsequent disease detection and semantic segmentation tasks. The preprocessing steps ensured that the model could accurately detect and segment diseased regions, thereby improving overall detection and segmentation performance. The combination of denoising, contrast enhancement, and color correction established a robust foundation for training deep learning models. In agricultural videos, challenges such as variable lighting conditions and complex backgrounds often complicate disease detection. The enhancement methods effectively addressed these challenges, enhancing the model’s robustness and accuracy. Thus, data enhancement not only played a pivotal role in improving model performance but also ensured model reliability in disease detection tasks conducted in the Bayannur region.

### 3.2. Proposed Method

**Figure 2:**
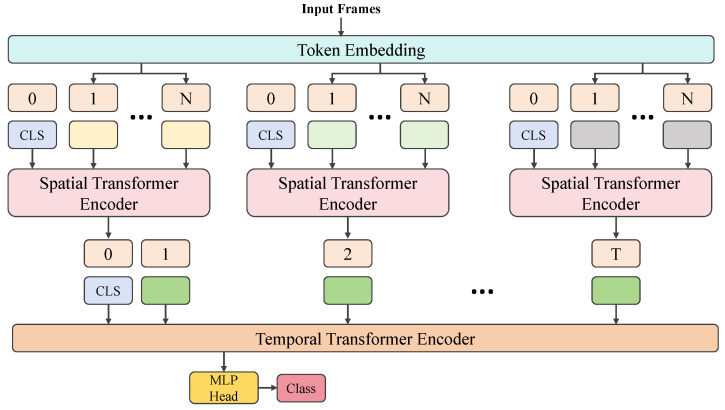
Overview of the proposed method in this paper.

A time-series segmentation network based on the Transformer model for tomato lesion segmentation is proposed, as shown in Figure 2. The model first processes the preprocessed image data using the Transformer architecture, leveraging the self-attention mechanism to capture global information in the image and enhance the segmentation accuracy of lesion regions. Subsequently, the maxmin-diffusion module is applied to enhance features, optimizing the saliency of the lesion areas and further improving segmentation accuracy. Next, the time-series segment network captures the temporal dependencies within video sequences, improving the model’s ability to handle dynamic changes in the lesion areas and increasing segmentation stability. Finally, the maxmin loss function module optimally adjusts the model output, ensuring accurate lesion area segmentation. The overall model effectively segments lesion areas in both static and dynamic video data through the collaborative functioning of these modules, providing an efficient and precise solution.

#### 3.2.1. Time-Series Segment Network Based on Transformer

**Figure 3:**
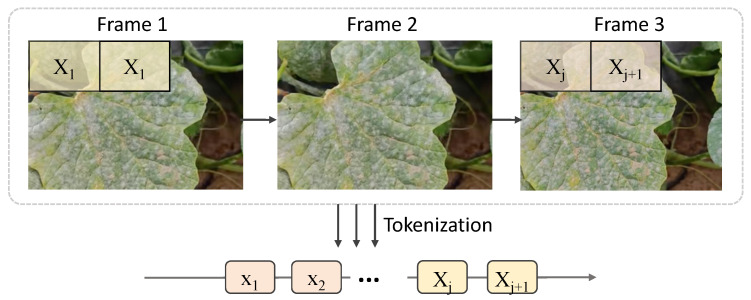
Process of video data.

This section details the specific implementation of the time-series segment network based on a Transformer, as proposed in this work, including network structure parameters, the distinction from traditional Transformers, and mathematical analysis that demonstrates the advantages of this design in the task at hand. Traditional Transformer networks are primarily designed for static, non-temporal input data. However, in time-series data processing, especially in video segmentation tasks, the temporal dynamics and the dependencies between frames are crucial for model performance. While traditional Transformers are capable of modeling long-range dependencies, they often lack effective temporal modeling when processing time-series data. To address this issue, an improved time-series segmentation network is proposed, which combines temporal information enhancement on top of the Transformer, specifically addressing how to effectively capture dynamic changes between time steps when processing video data, as shown in Figure 3.

**Figure 4:**
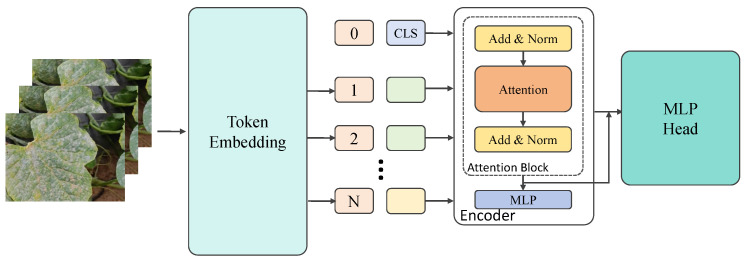
Architecture of time-series segment network based on Transformer.

Compared with traditional Transformers, there are two main differences in the time-series segmentation network proposed here. First, the preprocessing and representation of the input data differ. Traditional Transformers typically use a flattened input structure, but for video data, this work introduces temporal features and performs time-series modeling for the features of each frame in the video sequence, allowing the network to process both image features and temporal information. Second, specialized modules for time-series processing, such as the time-series segment network and the maxmin-diffusion module, are designed on top of the Transformer to more effectively capture the dynamic relationships between frames. In contrast, traditional Transformer networks are generally used for static tasks and do not have the capability to model such temporal dependencies. The time-series segmentation network proposed in this work consists of four main modules: the input feature extraction module, the Transformer encoder module, the time-series segmentation module, and the output prediction module. The overall network structure is shown in Figure 4, with each module playing a distinct role during processing, working in synergy to achieve lesion segmentation between video frames.

First, after data preprocessing, the feature of each video frame is passed into the network’s input feature extraction module. Each video frame is processed by a CNN to extract image features, which are then flattened into one-dimensional vectors for processing by the Transformer encoder. These image features are further combined with positional encoding to ensure that the network can leverage the sequential information from the time-series data. In the generation of positional encodings, sine and cosine functions are used to generate a position vector for each frame, and the specific formula is given as follows:

(14) $$PE\left(t,2i\right)=\sin\left(\frac{t}{{10,000}^{2i/d}}\right),\;PE\left(t,2i+1\right)=\cos\left(\frac{t}{{10,000}^{2i/d}}\right)$$

where *t* represents the time step, *i* denotes the dimension of the positional encoding, and *d* is the total dimensionality of the positional encoding. After merging this positional encoding with the input features, each frame’s features are provided with temporal sequence information, ensuring that the Transformer can understand the sequential relationship between frames. The features with positional encoding are then passed into the Transformer encoder module. Similar to traditional Transformer encoders, the encoder consists of multiple self-attention layers and feed-forward neural network layers. However, in the time-series segmentation network, in addition to the standard self-attention mechanism, an improved maxmin-diffusion module was designed that adjusts attention weights through a specialized mechanism to enhance the interaction between each time step in the time-series data, enabling the model to capture dynamic changes and temporal dependencies. Specifically, during the self-attention calculation in each layer, the query (*Q*), key (*K*), and value (*V*) matrices of the input features are first computed, and the similarity between these matrices is evaluated using the self-attention mechanism.

By weighting the similarity between input features, each feature in the output will consider the influence of other frames, thereby effectively capturing long-range dependencies in time-series data. In the maxmin-diffusion module, a diffusion mechanism is introduced that adjusts attention weights based on the principle of maximum and minimum differences. This mechanism allows for a more precise selection of which relationships between time steps are critical, reducing information redundancy and noise interference. The maxmin-diffusion module adjusts the weight of each time step, allowing the model to allocate more attention to critical moments in the lesion area while suppressing weights for irrelevant moments, further improving segmentation accuracy.

After processing through the Transformer encoder, the features are passed to the time-series segmentation module for further fusion and processing. The time-series segmentation module combines multi-scale feature fusion and temporal dynamic modeling, establishing stronger dependencies between each time step. The purpose of this module is to weight and merge the time-series features of each frame with the features of other frames to ensure that the prediction for each frame considers the dynamic changes across the entire video sequence. Finally, the processed features are passed to the output prediction module, which generates the final lesion segmentation results. This module typically includes a fully connected layer and a softmax classification layer, which are used to determine whether each time step’s features correspond to a lesion area. The final predictions are output as segmentation masks for each frame, indicating the lesion areas in the video.

From a mathematical perspective, the time-series segmentation network proposed in this work enhances temporal modeling on top of the traditional Transformer through the self-attention mechanism and the maxmin-diffusion module. Additionally, through the design of positional encoding, the model can fully utilize the temporal sequence information in video sequences, thus avoiding the issue in traditional Transformers where time sequences may be overlooked without positional encoding. The effective application of self-attention in time-series data ensures that the features of each frame consider not only the current frame’s information but also the contextual information from previous frames. This global dependency modeling approach offers significant advantages over traditional convolutional networks, enabling the handling of longer time-series dependencies.

#### 3.2.2. MaxMin-Diffusion Module

**Figure 5:**
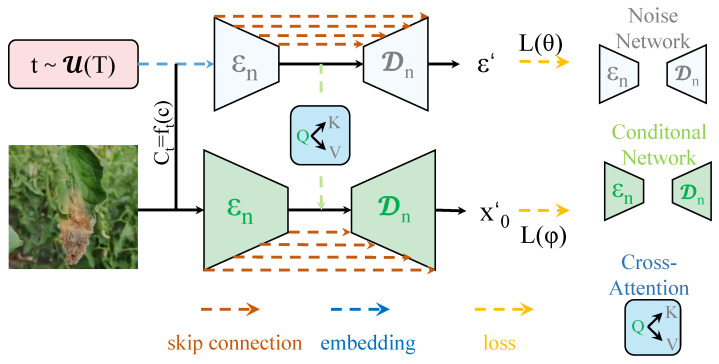
Architecture of maxmin-diffusion module.

The maxmin-diffusion module, a key innovation proposed in this work, builds upon the traditional diffusion module with the goal of enhancing the segmentation accuracy of lesion regions in temporal data for Transformer-based models. Unlike traditional diffusion modules, the main innovation of the maxmin-diffusion module lies in the introduction of the maxmin principle, which dynamically adjusts the attention weights within the model. This adjustment allows the model to prioritize frames with significant changes during the feature propagation process, optimizing the information transfer between time steps, as shown in Figure 5. Specifically, the maxmin-diffusion module performs weighted diffusion on each time step’s features, suppressing noise from less important moments while reinforcing the dependencies between key frames, thereby improving the model’s ability to handle temporal data.

The structural design of the maxmin-diffusion module draws inspiration from the self-attention mechanism in Transformer models, while incorporating traditional diffusion methods. The module consists of multiple layers, each containing a self-attention mechanism and a diffusion process to weight feature information. The overall design of the module is as follows:

1. Input layer: The input feature matrix is denoted as $X\in R^{N\times D}$, where *N* represents the number of time steps (i.e., the number of video frames), and *D* represents the feature dimension of each frame.
2. Maxmin-diffusion core module: In each layer, the query (*Q*), key (*K*), and value (*V*) matrices for each frame are computed, and their similarity is evaluated through the self-attention mechanism. Then, the attention weights between each pair of time steps are adjusted according to the maxmin principle. The calculation of this adjustment term is given by
  (15) $$\mathrm{MaxMin}\left(Q,K,V\right)=\frac{Q^{T}K}{{\left|Q^{T}K\right|}_{\max}-{\left|Q^{T}K\right|}_{\min}}$$
  Here, $Q^{T}K$ refers to the dot product of the query and key, and ${\left|Q^{T}K\right|}_{\max}$ and ${\left|Q^{T}K\right|}_{\min}$ represent the maximum and minimum values of the $Q^{T}K$ matrix, respectively. This approach allows the maxmin-diffusion module to prioritize the propagation of features between important time steps and suppress diffusion between irrelevant frames.
3. Output layer: After multiple diffusion processes, the output of the module is a new feature representation $X^{\prime }\in R^{N\times D}$, where the features of each time step have been optimized. The feature matrix $X^{\prime }$ is then passed to subsequent modules for further processing.

The design of the maxmin-diffusion module offers several notable mathematical advantages. First, traditional self-attention mechanisms typically determine the attention distribution by calculating the similarity between the query and key. In contrast, the maxmin-diffusion module, by incorporating the maxmin adjustment term, enables a more precise control of the information diffusion between time steps. In lesion segmentation tasks, this design ensures that the model pays higher attention to key frames and lower attention to irrelevant frames, thus enhancing segmentation accuracy. Moreover, the introduction of the maxmin principle allows the network to perform more flexible and fine-grained adjustments to the weighting of input features. In temporal data, there are often significant differences between crucial moments (e.g., moments of lesion change) and less important moments (e.g., background frames or frames with no lesions). Traditional self-attention mechanisms are unable to effectively recognize these differences, while the maxmin-diffusion module can dynamically adjust based on the maxmin principle, enabling more effective feature propagation. From a mathematical perspective, through maxmin adjustments, the model can assign higher attention weights to important moments and suppress the influence of irrelevant moments. This adjustment process reduces the propagation of redundant information in temporal data, ultimately improving the model’s ability to locate lesion regions. Specifically, when applied to lesion segmentation tasks, the maxmin-diffusion module is more precise in capturing the dynamic changes of the lesion regions compared to traditional diffusion modules, thereby improving the final segmentation results.

#### 3.2.3. MaxMin Loss Function

The maxmin loss function, an innovative loss function proposed in this work, offers superior handling of the imbalance problem in temporal data compared to traditional loss functions. It further enhances the model’s performance on dynamic video data. Traditional loss functions, particularly cross-entropy loss, typically measure model performance by calculating the difference between predicted and ground truth values. These loss functions are effective for optimizing model outputs in most scenarios, but their limitations become apparent when dealing with temporal data and dynamic changes. The maxmin loss function, by introducing a mechanism based on the maxmin principle, enables a more refined assessment of the importance of each temporal point. The core idea of the maxmin loss function is to adjust the loss value of each time step using the maximum and minimum differences, optimizing the training process of the model. Let $\hat{y_{t}}$ and $y_{t}$ denote the predicted and ground truth values at time step *t*, respectively. The traditional loss function often directly calculates and sums or averages the losses across all time steps, while the maxmin loss function adjusts the weight of the loss values by incorporating the maxmin principle. Specifically, the maxmin loss function is defined as

(16) $$L_{\mathrm{maxmin}}=\frac{1}{T}\sum_{t=1}^{T}\left|\hat{y_{t}}-y_{t}\right|\cdot \frac{1}{\left(\max\left(\hat{y_{t}},y_{t}\right)-\min\left(\hat{y_{t}},y_{t}\right)\right)+\epsilon }$$

where *T* represents the total number of time steps, $\hat{y_{t}}$ is the predicted value at time step *t*, and $y_{t}$ is the ground truth value. $\max(\hat{y_{t}},y_{t})$ and $\min(\hat{y_{t}},y_{t})$ represent the maximum and minimum values between the predicted and ground truth values, respectively, and ϵ is a small constant added to avoid division by zero. This design enables the maxmin loss function to emphasize the differences between time steps, enhancing the attention on critical moments while suppressing the loss from less important moments. The maxmin loss function works in close conjunction with the maxmin-diffusion module, and together, they influence the model’s training process. The maxmin-diffusion module propagates information across temporal data via a diffusion mechanism, while the maxmin loss function adjusts the weight of each time step’s loss during training, ensuring that the model focuses more effectively on the lesion regions at key moments. During the forward pass, the maxmin-diffusion module uses a self-attention mechanism and the maxmin principle to weight and propagate features, thereby giving more attention to the critical time steps. Simultaneously, during the backward pass, the maxmin loss function further strengthens the impact of key frames through weighted adjustments of the loss. Specifically, the maxmin loss function weights the loss at each temporal point to ensure that the model’s errors at key frames receive more correction, while errors at less important frames are adjusted to a lesser extent. This complementary interaction between the maxmin loss function and the maxmin-diffusion module enables the model to more accurately capture the dynamic changes in lesion regions, thereby improving the final segmentation accuracy.

The maxmin loss function demonstrates its unique advantages in the lesion segmentation task presented in this work. First, the changes in lesion regions in temporal data are often localized and brief, and traditional loss functions may fail to capture these dynamic changes effectively. By weighting the key frames, the maxmin loss function allows the model to focus more on the changes in the lesion regions, thereby improving segmentation accuracy. Second, the dynamic adjustment of losses at each temporal point makes the training process more efficient. Compared to traditional loss functions, the maxmin loss function reduces the computational burden from irrelevant moments, enhancing the efficiency of model training. Additionally, the maxmin loss function effectively mitigates the interference from background noise, allowing the model to segment the lesion regions more accurately.

### 3.3. Experimental Setup

#### 3.3.1. Hardware and Software Platforms

The hardware platform was critical in ensuring efficient training and the inference of deep learning models. To meet the demands of large-scale video data processing, high-performance GPUs were utilized for acceleration. Specifically, NVIDIA A100 Tensor Core GPUs were employed, renowned for their superior performance in deep learning tasks, particularly with large datasets and complex models. The A100 GPU, equipped with 40 GB of high-bandwidth memory, facilitated the efficient storage and processing of extensive image data, crucial for real-time video processing and training. The CPU utilized was the AMD EPYC 7742 processor, featuring 64 physical cores and 128 threads, providing robust computational support during large-scale data preprocessing and model training. The system was further equipped with 256 GB of DDR4 RAM, ensuring rapid data access and processing while minimizing memory bottlenecks during training. Additionally, SSD-based high-speed storage was employed to enhance data read and write speeds, reducing data loading delays and expediting the overall training process.

On the software side, PyTorch was selected as the primary deep learning framework due to its flexibility, efficiency, and support for both dynamic and static computation graphs. PyTorch’s modular design and simple API facilitated the development of video processing and image segmentation tasks. CUDA 11.3 and cuDNN 8.2 were integrated to fully exploit the computational capabilities of the A100 GPUs. CUDA 11.3, optimized for the latest GPU architectures, leveraged Tensor Core parallelism to accelerate model training significantly. cuDNN, a GPU-accelerated library for deep neural networks, enhanced convolutional operations, improving inference speed and accuracy, particularly in image segmentation and video analysis tasks. Python 3.8 was used for implementation, supplemented with libraries such as NumPy 1.24.0 and OpenCV 3.4.9.33 for efficient data preprocessing and augmentation. This combination of hardware and software platforms enabled the efficient processing of large video datasets, ensuring model accuracy and practical applicability.

#### 3.3.2. Hyperparameter Settings and Dataset Separation

The choice of optimizer and hyperparameter configuration was pivotal in ensuring efficient convergence and optimal performance of the deep learning model. For the tomato lesion segmentation task, the Adam optimizer (Adaptive Moment Estimation) was selected due to its capability to combine momentum and adaptive learning rates. Adam is particularly effective in handling sparse gradients and large-scale data, eliminating the complexity of manual learning rate adjustment. The update formula for the Adam optimizer is defined as

(17) $$\theta_{t}=\theta_{t-1}-\frac{\alpha }{\sqrt{v_{t}}+\epsilon }\cdot m_{t},$$

where $\theta_{t}$ represents the parameter at the *t*th update, $m_{t}$ and $v_{t}$ are the moving averages of the gradient and the squared gradient, respectively, α is the learning rate, and ϵ is a small constant used to prevent division by zero. By incorporating first- and second-moment estimates, Adam effectively adapts to sparse gradients and non-stationary objectives, making it suitable for tasks like video segmentation. For learning rate selection, an initial learning rate of $1\times 10^{-4}$ was chosen based on empirical tuning. A learning rate decay strategy was implemented, gradually reducing the learning rate as training progressed to avoid overfitting and premature convergence to local optima. Specifically, the learning rate was reduced by a factor of 0.1 every 50 epochs. Batch size was set to 16 to balance computational efficiency and memory constraints. Larger batch sizes accelerate training but increase memory consumption, while smaller batch sizes conserve memory at the expense of stability. Based on experimental results, a batch size of 16 was determined to be optimal for this task. The model was trained for a total of 200 epochs, providing sufficient iterations to capture the dataset’s complexity while ensuring effective model fitting. An early stopping strategy was applied, terminating training if validation loss ceased to decrease, thus mitigating overfitting. To further validate the model’s stability and generalization capability, 5-fold cross-validation was employed. The dataset was randomly divided into five subsets, with four subsets used for training and the remaining subset for validation in each fold. This approach ensured robust performance evaluation and reduced overfitting by testing the model across diverse data splits. The final performance metric was the average of the results from the five folds.

For dataset splitting, 80% of the data were allocated for training, while the remaining 20% were reserved for testing. Stratified sampling was utilized to maintain consistent class distributions between training and testing subsets, enhancing training stability and effectiveness. These optimization and hyperparameter settings ensured efficient training while maximizing the model’s segmentation accuracy and robustness. Proper learning rate scheduling, batch size adjustment, and cross-validation contributed to the model’s ability to generalize across diverse datasets, providing a reliable evaluation framework.

#### 3.3.3. Baselines

To evaluate the performance of the proposed tomato lesion segmentation model, several traditional semantic segmentation and time-series models were selected as baselines. Among traditional semantic segmentation models, DeepLab [40] and PSPNet [41] were chosen due to their widespread application in image segmentation, particularly in scenarios with complex backgrounds. DeepLab employs dilated convolutions to expand the receptive field, enabling the capture of rich contextual information and enhancing segmentation precision. PSPNet leverages a pyramid pooling module to aggregate multi-scale contextual information through pooling operations at varying scales, improving performance in complex scenes. DeepLab’s core advantage lies in its ability to capture extensive contextual information by increasing the dilation rate of convolutional kernels, while PSPNet excels in fusing global information through multi-scale pooling. Additionally, time-series models were included as baselines to evaluate the handling of dynamic changes in lesion progression. LSTM [42] and GRU [43] were selected for their capability to model sequential dependencies. LSTM addresses the vanishing gradient problem in long sequences through the use of forget, input, and output gates, effectively capturing temporal dynamics in video frames. These traditional semantic segmentation and time-series models provided performance references, enabling a comprehensive assessment of the proposed model’s effectiveness in tomato lesion segmentation tasks. Comparative analysis with these baselines highlighted the advantages and limitations of the proposed model in handling complex agricultural video data, offering insights for future improvements.

#### 3.3.4. Evaluation Metrics

In image segmentation tasks, particularly in practical applications such as tomato lesion segmentation, selecting appropriate evaluation metrics is essential for comprehensively assessing model performance. To evaluate the proposed lesion segmentation model, four commonly used metrics were employed: mean intersection over union (mIoU), precision, recall, and accuracy. These metrics not only quantify the model’s segmentation accuracy but also reflect its inference speed and practical efficiency.

The mIoU is calculated based on the intersection and union between the segmentation results and the ground truth label regions. Essentially, it measures the overall performance of the model by calculating the intersection over union (IoU) for each class and taking the average of the IoU values across all classes. Specifically, the IoU is the ratio of the area of the intersection of the predicted region and the ground truth region to the area of their union. The larger the IoU value, the better the model’s performance in that class. When calculating across multiple classes, the mIoU is the average of the IoU values for each class, providing a comprehensive reflection of the model’s segmentation performance across all classes. Precision is a metric used to measure the proportion of correctly predicted positive samples in all predicted positive samples. In the case of lesion segmentation tasks, precision is used to evaluate the proportion of actual lesions in the predicted lesion areas. Recall is a metric that measures the model’s ability to recognize the actual lesion regions. Recall refers to the proportion of successfully predicted lesion positive samples out of all actual lesion samples. Accuracy is a metric commonly used to measure the correctness of the model’s predictions. Accuracy refers to the proportion of correctly classified samples out of the total number of samples. The mathematical formulas for these metrics are as follows

(18) $$\mathrm{IoU}=\frac{\left|A\cap B\right|}{\left|A\cup B\right|}$$

(19) $$\mathrm{mIoU}=\frac{1}{N}\sum_{i=1}^{N}\frac{|A_{i}\cap B_{i}|}{|A_{i}\cup B_{i}|}$$

(20) $$\mathrm{Precision}=\frac{TP}{TP+FP}$$

(21) $$\mathrm{Recall}=\frac{TP}{TP+FN}$$

(22) $$\mathrm{Accuracy}=\frac{TP+TN}{TP+FP+TN+FN}$$

where *A* represents the predicted lesion region, *B* represents the ground truth lesion region, |A∩B| represents the intersection area of the predicted region and the ground truth region, and |A∪B| represents the union area of the predicted region and the ground truth region. *N* is the number of classes, and $A_{i}$ and $B_{i}$ are the predicted and ground truth regions for the *i*-th class, respectively. FN stands for False Negatives, TN for True Negatives, TP for True Positives, and FP for False Positives. In conclusion, the evaluation metrics chosen in this study—mIoU, precision, recall, and accuracy—allow for a comprehensive assessment of the lesion segmentation model from different dimensions.

## 4. Results and Discussion

### 4.1. Description of Video Data in Experiments

While the disease itself remains unchanged over short time periods, issues such as image blurring and significant spatial shifts caused by drone-based video collection are precisely one of the core motivations behind the design of the proposed method. Videos captured by drones may not exhibit noticeable disease dynamics over short temporal spans (e.g., a few seconds), but the rapid movement of drones results in substantial spatial variations. The inter-frame feature consistency and spatial changes across video frames can provide valuable information for disease detection. Specifically, the following two points highlight the importance of inter-frame information:

1. Supplementary Information Between Frames: In drone-captured videos, individual frames may suffer from issues such as unfavorable lighting conditions, occlusions, or focus changes, leading to blurred or missing local features. However, adjacent frames often contain complementary detail information. The proposed maxmin-diffusion module integrates spatial information from adjacent frames through an inter-frame diffusion mechanism, dynamically adjusting attention weights to prioritize critical frames, thereby improving the segmentation performance of disease regions. For instance, a specific frame may be blurred due to camera motion, but inter-frame correlations from adjacent frames can provide clearer features, ensuring the completeness and accuracy of the detected disease regions.
2. Spatial Consistency Modeling: The movement of drones introduces significant scene changes, such as perspective shifts and the displacement of plant regions. These spatial variations may prevent single-frame models from capturing the entirety of the disease region. By leveraging the time-series segmentation network, the proposed method can capture the spatial consistency features across video frames, such as the continuity of disease spots or the dynamic changes in leaf shapes. This capability effectively enhances the robustness and overall performance of segmentation, especially in scenarios with significant spatial variability.

### 4.2. Tomato Disease Detection Results

The design of the experiment aims to compare the performance of various deep learning models in disease detection tasks, particularly analyzing performance across multiple metrics, such as precision, recall, accuracy, and mIoU, for different model architectures and mechanisms. Specifically, LSTM-SegNet, GRU-SegNet, PSPNet, U-Net, DeepLab v3, DeepLab v3+, and the proposed maxmin-diffusion module-based model were selected for comparison. The experimental results of these models, as shown in Table 2, allow for an in-depth exploration of the performance differences of various neural network architectures when handling disease detection tasks. These results, combined with an analysis of their mathematical and algorithmic characteristics, further reveal the reasons behind the observed model performance variations.

From the experimental results, it can be observed that all models show differences in precision, recall, accuracy, and mIoU. The LSTM-SegNet model achieves a precision of 0.82, recall of 0.78, accuracy of 0.80, and mIoU of 0.79, which is relatively conservative compared to the other models. LSTM networks are known for their strong memory capabilities when processing sequential data, but when it comes to handling complex image segmentation tasks, particularly involving fine details of diseases, the recursive structure of an LSTM network can lead to the attenuation of information flow. This is especially evident in areas with dense temporal features, where fine details may be overlooked. Additionally, the vanishing or exploding gradient problem during training can restrict the performance of LSTM models, leading to suboptimal results in complex tasks. The GRU-SegNet model shows improvements in precision, recall, accuracy, and mIoU, with values of 0.85, 0.81, 0.83, and 0.82, respectively. The GRU optimizes the LSTM structure by reducing parameter count and computational complexity, making the training process more efficient. Furthermore, the gating mechanism in the GRU helps mitigate the vanishing gradient problem, allowing for the retention of important temporal information. However, despite these improvements, the GRU still faces limitations, particularly in modeling spatial features when processing large-scale image data. This results in subpar performance in precise segmentation and detail capturing when compared to more advanced models. PSPNet achieves further improvement in precision, recall, accuracy, and mIoU, with values of 0.88, 0.84, 0.86, and 0.85, respectively. PSPNet introduces a pyramid pooling module that better handles multi-scale feature information and enhances segmentation accuracy through the integration of global context. Its complex structure enables the extraction of useful features from different scale contexts, which is significant for the segmentation of disease regions. However, PSPNet is computationally expensive, and when handling the spatiotemporal variations in complex disease areas, the model’s expressive power may be insufficient, especially for fine-grained classification and small-object detection. DeepLab v3 and DeepLab v3+ are state-of-the-art models in semantic segmentation, with precision and recall values of 0.89 and 0.86 and 0.87 and 0.87, respectively, demonstrating strong spatial feature extraction abilities. Both DeepLab models utilize dilated convolutions to expand the receptive field without increasing computational cost, capturing more extensive contextual information. This makes DeepLab v3 and v3+ excel in handling large-scale and high-resolution images, particularly in the extraction of global information for complex disease regions. DeepLab v3+ further improves on DeepLab v3 by adding an encoder–decoder structure, which enhances spatial detail recovery. Although it performs well in segmentation tasks, its complex structure demands significant computational resources. The proposed maxmin-diffusion model outperforms all other models in terms of precision, recall, accuracy, and mIoU, with values of 0.94, 0.90, 0.92, and 0.91, respectively. By introducing a maximum–minimum difference-based attention mechanism, the maxmin-diffusion module optimizes the segmentation precision of disease regions in sequential data. Compared to traditional models, maxmin-diffusion more effectively captures spatiotemporal changes between key frames and suppresses noise from irrelevant frames through a weighted diffusion mechanism, enhancing the dependencies between key frames. This allows the model to focus on frames with significant changes, thereby improving the localization and segmentation of disease areas. Mathematically, the maxmin-diffusion model uses a maximum–minimum difference adjustment term to precisely control the propagation of information when computing attention weights for each time step, thus avoiding interference from irrelevant information. This mechanism enables the model to capture key features more accurately in the dynamic changes of disease regions, leading to significantly improved segmentation performance.

The results indicate that the performance differences across models in disease detection tasks primarily stem from their structural design and information processing mechanisms. Traditional LSTM and GRU networks, which rely on the transmission of sequential information, are effective in handling temporal data. However, their spatial feature modeling capabilities are relatively weak, which limits the performance of these models in more complex disease regions. PSPNet and DeepLab series excel in spatial feature extraction but lack efficient temporal modeling capabilities, making them less effective when handling dynamic disease regions. In contrast, the maxmin-diffusion model introduces innovations in processing sequential data by employing the maximum–minimum difference principle, which allows for a more effective capture of dynamic changes in disease regions, resulting in a significant improvement in segmentation accuracy. Mathematically, the maxmin-diffusion module enhances feature propagation between key frames by dynamically adjusting attention weights and suppressing the influence of irrelevant frames, thus improving the overall performance of the model.

### 4.3. Result Analysis

The objective of this experiment was to conduct a detailed analysis of the detection performance for different types of diseases, evaluating the model’s precision, recall, accuracy, and mIoU metrics when handling various diseases. This analysis not only helps validate the superiority of the maxmin-diffusion model in disease detection tasks but also provides a theoretical basis for the detection of different disease types, aiding in the further optimization of the model’s application in the agricultural field.

From the experimental results in Table 3, the detection performance for gray mold is relatively ideal, with a precision of 0.90, recall of 0.87, accuracy of 0.88, and mIoU of 0.88. This indicates that the model is effective at identifying disease regions and achieving high detection accuracy for gray mold. However, the recall rate is slightly lower than precision, suggesting that in some cases, the model may miss certain disease regions, especially in areas where the background or symptoms are not as distinct. The detection performance for late blight shows improvement, with a precision of 0.91, recall of 0.89, accuracy of 0.90, and mIoU of 0.90. This result further confirms the advantages of the maxmin-diffusion model in detecting more complex diseases, particularly in accurately identifying disease boundaries and fine details. The detection performance for Early Blight is slightly higher than that of late blight, with a precision of 0.94, recall of 0.90, accuracy of 0.92, and mIoU of 0.91. The higher performance for this disease may be attributed to the stronger contrast between the disease regions and the background, which allows the model to more precisely extract and segment disease areas. The detection results for viral disease are outstanding, with a precision of 0.96, recall of 0.92, accuracy of 0.94, and mIoU of 0.93, showing that the model is capable of handling the complex features of the disease, especially under varying backgrounds, while maintaining a high level of segmentation capability. The detection performance for bacterial spot is the best, with a precision of 0.98, recall of 0.95, accuracy of 0.96, and mIoU of 0.96. This suggests that the model has extremely high accuracy and reliability for detecting bacterial spot, which could be due to the clearer characteristics of the disease region in the images and the more concentrated spatial distribution of this disease, enabling the model to effectively extract features and perform precise segmentation in this scenario.

### 4.4. Ablation Study of Different Feature Extraction Mechanisms

The aim of this experiment was to evaluate the impact of different feature extraction mechanisms on disease detection performance through an ablation study, with the goal of revealing the contribution of the maxmin-diffusion mechanism in optimizing Transformer models. By comparing it with other common feature extraction mechanisms such as self-attention and CBAM, the experiment sought to explore how different attention mechanisms affect the segmentation of disease regions and analyze the advantages and limitations of these mechanisms in the image feature extraction process from both a mathematical and theoretical perspective.

From the experimental results shown in Table 4, it can be observed that the self-attention mechanism performs the worst, with a precision of 0.76, recall of 0.71, accuracy of 0.73, and mIoU of 0.74. This indicates that while the self-attention mechanism can capture long-range dependencies in the image, it has significant limitations in fine-grained feature extraction and precise localization of regions, resulting in lower segmentation accuracy for disease regions. Self-attention relies solely on global information from the input features, and when handling disease areas with complex backgrounds or similar characteristics, it is prone to interference, leading to misclassifications or missed detections. In contrast, the CBAM mechanism shows improvement, with a precision of 0.85, recall of 0.82, accuracy of 0.84, and mIoU of 0.84. By introducing channel attention and spatial attention modules, CBAM can simultaneously consider the dependencies between channels and the importance of spatial regions during feature extraction, thus enhancing the model’s focus on disease regions. This dual-attention mechanism allows the model to more accurately extract disease features, particularly when spatial region characteristics are more distinct. However, although CBAM has significant advantages in improving disease detection performance, it still lacks special handling for the dynamic changes in sequential data, which limits its optimal segmentation performance in complex dynamic scenarios. The maxmin-diffusion mechanism achieves the best performance, with a precision of 0.94, recall of 0.90, accuracy of 0.92, and mIoU of 0.91, indicating its significant advantages in handling sequential data with dynamic changes. Maxmin-diffusion dynamically adjusts the attention weights by introducing the maximum–minimum difference principle, effectively strengthening the dependencies between key frames while suppressing the interference from irrelevant frames, thus optimizing the segmentation accuracy of disease regions. Mathematically, the maxmin-diffusion module finely adjusts attention at each step through the maximum–minimum difference, allowing the model to more effectively suppress redundant information and enhance its responsiveness to fine-grained changes at critical moments in the sequential data. This mechanism fundamentally solves the issues of excessive information diffusion and uneven attention distribution inherent in traditional attention mechanisms, thereby demonstrating superior performance in dynamic disease detection tasks.

### 4.5. Ablation Study of Different Loss Functions

The aim of this experiment was to evaluate the impact of different loss functions on the performance of disease detection models, particularly the advantages of maxmin loss in optimizing disease region segmentation accuracy. By comparing it with common loss functions such as cross-entropy loss and focal loss, the experiment sought to reveal the contribution of each loss function to model training, especially in optimizing key metrics like precision, recall, and mIoU when handling imbalanced datasets.

From the experimental results shown in Table 5, the model using cross-entropy loss performs the worst, with a precision of 0.68, recall of 0.65, accuracy of 0.67, and mIoU of 0.66. This indicates that, although cross-entropy loss is the most commonly used classification loss function, it is not ideal for tasks involving imbalanced classes or complex regions. Cross-entropy loss does not adequately account for class imbalance, which results in the model having lower recall and accuracy when handling disease regions of minority classes, thus reducing overall performance. In contrast, the model using focal loss shows improved performance, with a precision of 0.84, recall of 0.80, accuracy of 0.82, and mIoU of 0.82. Focal loss addresses class imbalance by assigning higher weights to harder-to-classify samples, enabling the model to focus more on difficult-to-classify disease regions, particularly those in the background or easily classified areas. While focal loss improves model performance in the presence of class imbalance, it still fails to capture fine-grained changes in disease region details and may have limited performance when handling dynamically changing sequential data. In comparison, the maxmin loss shows the best performance, with a precision of 0.94, recall of 0.90, accuracy of 0.92, and mIoU of 0.91. This demonstrates that maxmin loss not only effectively addresses class imbalance but also optimizes disease region segmentation accuracy by finely adjusting the model’s attention to key regions through the maximum-minimum difference adjustment mechanism. Mathematically, maxmin loss dynamically adjusts the loss function based on the maximum–minimum difference for each time step, allowing the model to focus more on key disease change areas while reducing the impact of redundant information. This mechanism improves the model’s performance in sequential data, enabling more accurate localization of disease regions, and shows superior performance, particularly in dynamic disease detection tasks.

### 4.6. Model Lightweight Deployment

To ensure that the disease detection model proposed in this paper can run efficiently on mobile devices (such as Huawei Mate 60 is manufactured by Huawei in Dongguan, China), a lightweight model deployment approach was implemented. The goal was to achieve high-precision disease detection despite the limitations of computational resources and memory. The lightweighting process began with network architecture optimization to improve inference speed by reducing computational complexity and memory usage. To this end, lightweight techniques such as depthwise separable convolution and pruning were employed, which effectively reduced the model’s computational load and storage requirements. Depthwise Separable Convolution splits the traditional convolution operation into two stages: the first stage applies convolution independently to each input channel, and the second stage applies pointwise convolution to the convolution results. This reduces the computation from the conventional convolution formula C×K×K×W×H to C×K×K×W+C×K×K×H, significantly lowering the consumption of computational resources. Additionally, pruning removes unimportant neuron connections or neurons to further reduce the complexity of the model, making it more lightweight and better suited to low-power devices. During deployment, the model was first quantized. Quantization converts floating-point operations into fixed-point operations, which reduces both the model’s storage requirements and computational precision loss. The specific formula for quantization is

(23) $$\hat{w}=\mathrm{round}\left(\frac{w}{\Delta_{w}}\right)$$

where $\hat{w}$ is the quantized weight value, *w* is the original weight value, and $\Delta_{w}$ is the quantization step size. With this method, the quantized weights can be represented with fewer bits. The quantized model significantly reduces storage space, decreases memory usage, and accelerates the inference process, offering strong advantages when deployed on mobile devices. Furthermore, to further enhance inference speed, the knowledge distillation technique was employed. This involves transferring the knowledge from a complex model to a smaller student model, retaining the accuracy of the large model while reducing the computational load. In the distillation process, the output of the teacher model serves as the target for the student model, and knowledge transfer is achieved by minimizing the Kullback–Leibler (KL) divergence between their outputs. The formula for this is

(24) $$L_{KD}=\sum_{i}p_{t}\left(x_{i}\right)\log\left(\frac{p_{t}\left(x_{i}\right)}{p_{s}\left(x_{i}\right)}\right)$$

where $p_{t}$ and $p_{s}$ are the output distributions of the teacher and student models, respectively, $x_{i}$ is the input sample, and $L_{KD}$ is the knowledge distillation loss function. Using this method, the student model can approximate the performance of the teacher model at a lower computational cost, enabling efficient deployment and inference. Finally, after a series of optimizations and compression operations, the model in this paper was successfully deployed on the Huawei Mate 60 mobile terminal, achieving efficient disease detection that meets both real-time and accuracy requirements.

### 4.7. Discussion

This study proposed a tomato disease detection model based on the maxmin-diffusion mechanism and time-series network, and its superiority in dynamic data analysis and complex background processing was validated through experiments. To further highlight the contributions of this study in the field, this section compares our approach with other related studies and supplements the discussion with references to enhance its depth and comprehensiveness.

First, compared to the tomato leaf disease detection system based on an SVM proposed by Rahman et al. [22], our model demonstrates significant advantages in detection accuracy and the ability to handle complex scenarios. Although Rahman et al.’s system achieved an accuracy of 85%, it heavily relies on traditional machine learning models and feature extraction, making it less effective in dealing with complex backgrounds and detecting small lesions. By introducing the maxmin-diffusion mechanism, our approach effectively suppresses noise interference in complex backgrounds and enhances focus on lesion areas, thereby improving segmentation precision and overall detection performance. Second, compared to the Reconstructed Deep Residual Dense Network proposed by Zhou et al. [23], our model shows greater innovation and applicability in capturing temporal features. While Zhou et al.’s method achieved a detection accuracy of 95% in static image-based disease detection, it does not leverage the temporal correlation present in video sequence data, making it inadequate for handling the dynamic progression of diseases over time. The time-series network designed in our study effectively learns temporal features from video sequences, combining spatial and temporal information to improve the understanding of disease progression dynamics and further enhance detection accuracy. Additionally, compared to the study by Agarwal et al., which utilized an improved YOLOv3 algorithm [24], our approach demonstrates advantages in segmentation accuracy and detection efficiency. Agarwal et al.’s method achieved a detection accuracy of 92.39% through multi-scale feature detection and object bounding box clustering, but it is primarily focused on object detection rather than fine-grained disease segmentation. Our model, built on the maxmin diffusion mechanism and integrated with the maxmin loss function, enables the precise segmentation of lesion areas, demonstrating superior robustness in detecting small lesions and handling background interference.

Moreover, the advantages of the maxmin-diffusion mechanism and the time-series network in suppressing background noise and capturing temporal features are further supported by relevant theoretical and experimental studies. For example, the global feature propagation capability of diffusion mechanisms has been validated in multiple studies, and the temporal feature capture ability of the Transformer framework has been widely applied in other dynamic data processing fields. These references provide a solid theoretical foundation and practical support for the methods proposed in this study. In summary, the proposed method outperforms existing approaches in terms of accuracy, efficiency, and the ability to analyze dynamic disease progression. By integrating the maxmin diffusion mechanism, time-series network, and maxmin loss function, our method effectively handles complex backgrounds and small lesions while capturing the temporal evolution of diseases, making it highly valuable for practical applications. Future research will further explore optimizing the model structure to reduce computational costs and expand its applicability to other crop disease detection domains.

## 5. Conclusions

This paper proposes a disease detection model based on the maxmin-diffusion mechanism and explores its application in the agricultural field. With the advancement of agricultural automation, disease detection has become an essential task in smart agricultural systems. Traditional models face challenges in terms of accuracy and robustness when dealing with complex disease types. In this paper, we introduce the maxmin-diffusion mechanism to optimize the performance of the Transformer model in fine-grained feature extraction and sequential data processing. This mechanism dynamically adjusts attention weights, enhances focus on key regions, and suppresses interference from irrelevant areas, thereby improving the segmentation accuracy of disease regions. Experimental results demonstrate the superior performance of the maxmin-diffusion model across various disease detection tasks. For example, for gray mold, the detection accuracy was 0.90, recall was 0.87, accuracy was 0.88, and mIoU was 0.88; for bacterial spot disease, the detection accuracy was 0.98, recall was 0.95, accuracy was 0.96, and mIoU was 0.96, indicating that the model can accurately identify disease regions even in complex backgrounds. Moreover, comparison experiments with self-attention and CBAM mechanisms show that the maxmin-diffusion mechanism excels in extracting detailed features of disease regions, especially in the context of dynamic sequential data. To enable efficient deployment on mobile devices, this study employed lightweight techniques such as depthwise separable convolution, pruning, quantization, and knowledge distillation, significantly reducing the model’s computational and storage requirements. As a result, the model can perform efficient inference on low-power devices like the Huawei Mate 60, meeting both real-time and accuracy requirements.

## Figures and Tables

**Table 1:** Number of images for each disease.

Disease	Number of Images
Gray Mold	1000
Early Blight	1200
Late Blight	1500
Viral Disease	900
Bacterial Spot Disease	1800

**Table 2:** Tomato disease detection results.

Model	Precision	Recall	Accuracy	mIoU
LSTM-SegNet	0.82	0.78	0.80	0.79
GRU-SegNet	0.85	0.81	0.83	0.82
PSPNet	0.88	0.84	0.86	0.85
U-Net	0.90	0.88	0.89	0.90
DeepLab v3	0.89	0.86	0.87	0.87
DeepLab v3+	0.91	0.89	0.90	0.89
Proposed Method	0.94	0.90	0.92	0.91

**Table 3:** Detailed disease detection results.

Model	Precision	Recall	Accuracy	mIoU
Gray Mold	0.90	0.87	0.88	0.88
Late Blight	0.91	0.89	0.90	0.90
Early Blight	0.94	0.90	0.92	0.91
Viral Disease	0.96	0.92	0.94	0.93
Bacterial Spot	0.98	0.95	0.96	0.96

**Table 4:** Ablation study of different attention mechanisms.

Model	Precision	Recall	Accuracy	mIoU
Self-Attention	0.76	0.71	0.73	0.74
CBAM	0.85	0.82	0.84	0.84
MaxMin-Diffusion	0.94	0.90	0.92	0.91

**Table 5:** Ablation study of different loss functions.

Model	Precision	Recall	Accuracy	mIoU
Cross-Entropy Loss	0.68	0.65	0.67	0.66
Focal Loss	0.84	0.80	0.82	0.82
MaxMin Loss	0.94	0.90	0.92	0.91

## Data Availability

The data presented in this study are available on request from the corresponding author.
